# Lower Levels of Directed Exploration and Reflective Thinking Are Associated With Greater Anxiety and Depression

**DOI:** 10.3389/fpsyt.2021.782136

**Published:** 2022-01-07

**Authors:** Ryan Smith, Samuel Taylor, Robert C. Wilson, Anne E. Chuning, Michelle R. Persich, Siyu Wang, William D. S. Killgore

**Affiliations:** ^1^Laureate Institute for Brain Research, Tulsa, OK, United States; ^2^Department of Psychology, University of Arizona, Tucson, AZ, United States; ^3^Department of Psychiatry, University of Arizona, Tucson, AZ, United States

**Keywords:** directed exploration, random exploration, cognitive reflection, anxiety, depression, explore-exploit dilemma

## Abstract

Anxiety and depression are often associated with strong beliefs that entering specific situations will lead to aversive outcomes – even when these situations are objectively safe and avoiding them reduces well-being. A possible mechanism underlying this maladaptive avoidance behavior is a failure to reflect on: (1) appropriate levels of uncertainty about the situation, and (2) how this uncertainty could be reduced by seeking further information (i.e., exploration). To test this hypothesis, we asked a community sample of 416 individuals to complete measures of reflective cognition, exploration, and symptoms of anxiety and depression. Consistent with our hypotheses, we found significant associations between each of these measures in expected directions (i.e., positive relationships between reflective cognition and strategic information-seeking behavior or “directed exploration”, and negative relationships between these measures and anxiety/depression symptoms). Further analyses suggested that the relationship between directed exploration and depression/anxiety was due in part to an ambiguity aversion promoting exploration in conditions where information-seeking was not beneficial (as opposed to only being due to under-exploration when more information would aid future choices). In contrast, reflectiveness was associated with greater exploration in appropriate settings and separately accounted for differences in reaction times, decision noise, and choice accuracy in expected directions. These results shed light on the mechanisms underlying information-seeking behavior and how they may contribute to symptoms of emotional disorders. They also highlight the potential clinical relevance of individual differences in reflectiveness and exploration and should motivate future research on their possible contributions to vulnerability and/or maintenance of affective disorders.

## Introduction

Adaptive behavior requires striking an optimal balance between seeking desired outcomes and gathering information to best acquire those outcomes. The difficult task of finding this optimal balance is known as the explore-exploit dilemma. If one is confident in their current understanding of the world – and believes the contingencies in the world are stable – it makes sense to exploit current knowledge when making decisions to achieve one's goals. In contrast, if one is uncertain about the expected outcomes of different choices – or believes those outcomes can change over time – it is more adaptive to first gather more information. Research in psychology and neuroscience has produced a substantive literature characterizing both optimal and sub-optimal solutions to this dilemma that are exhibited by humans and other animals [reviewed in (1, 2)]. One common strategy proposed in the reinforcement learning literature is random exploration, in which the drive to exploit high-value options is countered by randomness in the decision process (3). Another strategy that derives from optimal solutions to the explore-exploit dilemma is directed exploration, in which decisions are biased toward more uncertain and/or more informative options (4, 5). By now, empirical evidence for the existence of both types of exploration in human behavior is overwhelming [e.g., (6–13)].

The importance of solving the explore-exploit dilemma appropriately has more recently motivated the hypothesis that one or more psychiatric disorders could be characterized by a poor balance in seeking information vs. reward (14). One possible imbalance involves under-exploration, in which an individual may show insufficient information-seeking behavior in novel situations. Under-exploration can promote avoidance of feared situations that are in fact safe and can hinder the opportunity to learn more adaptive patterns of behavior (15–17). Another possibility is over-exploration, in which an individual may remain uncertain for too long (perhaps because they do not learn from their experience) and fail to use current knowledge to efficiently seek out reward. Theoretically, these deficit profiles could be transdiagnostic or they could differ in different disorders. They could also be specific to random or directed exploration.

A few studies to date have begun to examine this hypothesis within substance use disorders, schizophrenia, and, to a lesser extent, emotional disorders [for a review, see (14)]. For example, one study found that, relative to healthy participants, individuals with alcohol use disorder (AUD) displayed less exploratory behavior in the context of both gains (i.e., trying to maximize reward) and losses (i.e., trying to minimize negative outcomes), while individuals with binge eating disorder showed greater exploration than those with AUD in the context of losses (18). Reduced exploration was also observed across all participants in the context of losses relative to gains. A second study in a heterogeneous population of substance use disorder (SUD) patients found no difference in directed exploration compared to healthy participants, but found that SUDs were associated with slower learning rates from losses and greater randomness in choice [however, this was not clearly tied to random exploration; (19)]. However, substance use may also influence exploration in the absence of any disorder. For example, nicotine smokers have also been found to make fewer exploratory choices and to possess higher learning rates relative to healthy participants (20); also see (21, 22).

Recent work in Schizophrenia patients found reduced directed exploration relative to healthy participants, while random exploration did not differ between groups (23). Exploratory behavior was not associated with negative symptoms, but random exploration was positively correlated with psychotic symptoms and cognitive impairment. Faster learning rates were also associated with more severe psychotic symptoms. Another recent study in healthy participants showed that normative levels of trait anxiety were positively associated with exploratory behavior and that an inverted-U pattern characterized performance differences; namely, moderate anxiety and associated levels of exploration led to higher levels of performance than those with very high anxiety or very low anxiety [i.e., who explored too much or too little; (24)]. This finding was in the context of a volatile environment with changing reward or loss probabilities and appeared to be driven by reduced reward-seeking behavior and an elevated drive to reduce uncertainty. This is consistent with previous work showing over-exploration in depression (25), and also consistent with suggestive evidence of greater exploration with higher anxiety and depression symptoms in one of the aforementioned studies on SUDs (19). However, greater anhedonia (in patients with Schizophrenia) has also been associated with reduced exploratory behavior in prior work (26), and a more recent study reported lower directed exploration in those with higher trait somatic anxiety (27). Therefore, while more research is needed to determine the exact nature and direction of relationships with specific symptoms/disorders, this growing body of work suggests that a range of potential differences in explore-exploit behavior may be present in the context of poor mental health – and could reflect state differences or vulnerability factors.

A further question pertains to the origins of variability in exploratory behavior. Normatively speaking, information-seeking should scale with estimates of one's own uncertainty. Therefore, under identical conditions, one would expect greater exploratory behavior in those who are more uncertain – yet this need not entail that this level of uncertainty is warranted given past observations. One may “jump to conclusions” and cease exploration prematurely, or uncertainty could persist despite having gathered sufficient information to infer the best course of action. One source of these differences in uncertainty estimation could be trait differences in cognitive reflection. This refers to the degree to which an individual tends to “go with their gut” and trust initial intuitions about how to respond vs. engaging effortful cognitive processes to reflect on the reliability of those intuitions. In recent years, such differences have been studied using performance measures such as the cognitive reflection test [CRT-7; (28)] and the comprehensive assessment of rational thinking [CART; (29)], as well with self-report measures of epistemic attitudes (30). However, these reflective tendencies have not been studied in the context of explore-exploit behavior, nor have they been thoroughly investigated in relation to psychopathology.

In this study, we first test the competing hypotheses that variation in symptoms of depression and/or anxiety in a community sample will be associated with higher vs. lower levels of exploratory behavior, and we examine whether directed vs. random exploratory strategies are affected. We then test the hypothesis that differences in explore-exploit behavior are predicted by differences in cognitive reflection. Finally, we examine whether cognitive reflection predicts symptoms of depression and/or anxiety and whether this relationship may be accounted for by differences in explore-exploit behavior.

## Methods

### Participants

A convenience sample of students at the University of Arizona as well as individuals from the surrounding community (mean age = 23.75, SD = 5.61 years; minimum age of 18), 115 male (79 students) and 301 female (206 students), was recruited from Tucson, AZ. Participants gave informed consent and were paid for their participation as part of a larger funded study. To motivate engagement, they were told they could win additional money based on task performance (up to an additional $50 across both the Horizon task included in this report [see below] and a second task not included here); however, all participants received the full $50 at the end of the study. This study was approved by the University of Arizona Institutional Review Board (Protocol #1607696724) and the Human Research and Protection Office of the U.S. Army Medical Research and Development Command (Protocol #A-19136.a and A-19136.b).

### Measures

#### Depression and Anxiety Symptoms

Participants completed the Beck Depression Inventory (BDI-II), a 21-item scale that measures symptoms of depression (31). Each item on the BDI is scored from 0 to 3, with higher scores consistent with depressive symptomatology. Participants also completed the state-trait anxiety inventory (STAI), a 40-item scale that measures anxiety symptoms in the present moment (state) and in life more generally (trait) (32). This data on depression and anxiety symptoms was also utilized in a recent study examining questions unrelated to exploration or cognitive reflection (33).

#### Levels of Directed and Random Exploration

**Figure 1:**
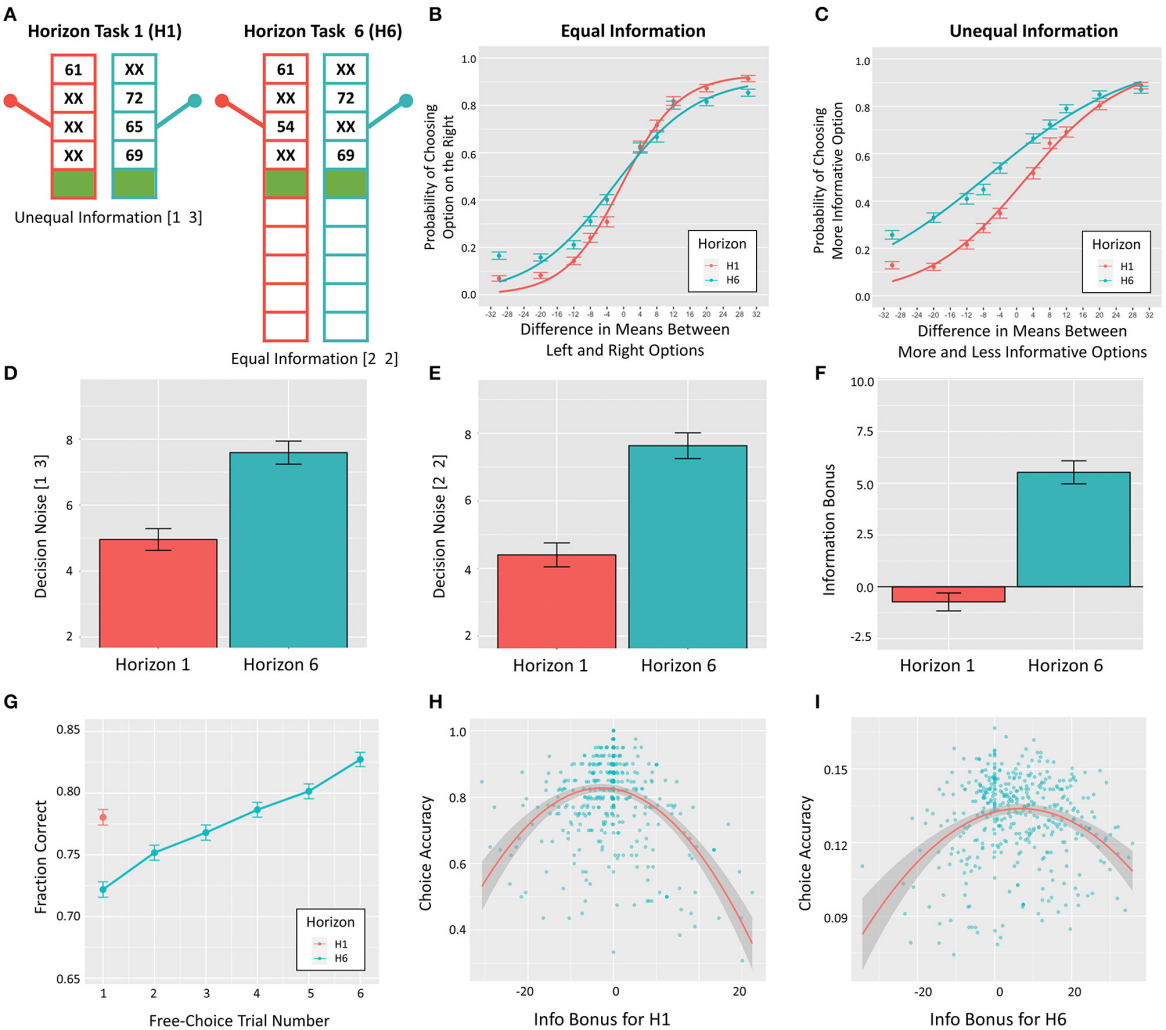
**(A)** Depiction of example Horizon 1 (H1) and Horizon 6 (H6) games at the first free choice. **(B,C)** Probability of choosing the option on the right depending on differences in the generative means of the two options. **(D,E)**. Estimates of decision noise in H1 and H6 games (within equal and unequal information conditions), showing the predicted pattern of greater noise in H6 games. **(F)** Estimates of information bonus in H1 and H6 games, showing the predicted pattern of greater information bonus values in H6 games. **(G)** Fraction of correct responses (i.e., choice of the option with the higher generative mean) for each trial in H1 and H6 games. **(H,I)** Inverted-U relationship between information bonus and choice accuracy (i.e., choice of the option with the higher true mean) in H1 and H6 games.

To measure directed and random exploration, participants completed a previously validated task called the Horizon Task (13). In this task, participants are asked to repeatedly choose between one of two options that can win the player an unknown number of points (see Figure 1A). In this paper, we used a version of the task with 80 games, where each game includes either 5 or 10 repeated choices (40 games of each length, interleaved). Each option can pay out between 1 and 100 points drawn from a Gaussian distribution (rounded to the nearest integer) with a fixed standard deviation of 8 points. The generative means of the underlying Gaussians are different for the two options and remain stable within a game. In each game, the mean of one option is set to either 40 or 60 points and the mean of the other is set such that the difference between the two means is 4, 8, 12, 20, or 30 points (counterbalanced across games with respect to left vs. right option and mean differences; see Figures 1B,C). Through a set of illustrated onscreen instructions, participants are explicitly told that the means of the two options are constant across trials within a game and that the variability in the values for both options is constant across all games. Participants are told that one option, in any given game, is always better on average, and they are instructed to maximize the points they earn.

In each game, the first four choices are forced (i.e., participants are told which option to choose). In “horizon 1” (H1) games they are then allowed to make one free choice, while in “horizon 6” (H6) games they are allowed to make six free choices (see Figure 1A). The slot machine display for each option shows the point value for the chosen option and an “XX” for the unchosen option, and outcomes of the whole sequence of choices remain on the screen throughout the game (i.e., each slot machine has either 5 or 10 blank boxes that are sequentially filled with each choice in the game; see Figure 1A). The forced choices could provide either equal information [two outcomes from each option (2 2)] or unequal information [one outcome from one option and three from the other (1 3)]. This setup allows assessment of the first free choice under equal vs. unequal information when participants expect to make one or six future choices. Previous studies using this task have shown that, during unequal information games, participants are more likely to choose the more uncertain option (i.e., where they have only seen a single outcome) in H6 games compared to H1 games (13). This is interpreted as a form of directed exploration in which the initial choice is expected to improve the ability to maximize reward on the subsequent five choices (i.e., whereas this would have no advantage in H1 games). In both equal and unequal information conditions, participants are also more likely to choose the option with the lower mean (of the forced choice outcomes) in H6 games than H1 games. This is interpreted as a form of random exploration, in which the lower mean choice also facilitates information gain and can benefit future choices (i.e., which would also have no advantage in H1 games).

Computational modeling is used to quantify individual differences in levels of directed exploration and random exploration based on the option chosen on the first free choice. In this model, the value *Q*_*a*_ of each option *a* is used to make probabilistic choices (i.e., a higher value leads to a higher probability of selection). The values of *Q*_*a*_ are computed as follows:


$$\begin{array}{l} Q_{a}=R_{a}+\alpha I_{a}+Bs_{a} \end{array}$$

Here, the experimental parameters are: *R*_*a*_ the expected reward, *I*_*a*_ the information value, and *s*_*a*_ the side on which option *a* is presented. Subject-specific parameters are: α, the value of information, which acts as an information bonus, and *B* the spatial bias. The relationship between *Q*_*a*_ and the probability of selecting each option is also influenced by decision noise within a logistic function with subject-specific variance parameter: σ_*d*_. The probability of choosing option *a* over option *b* is then:


$$\begin{array}{l} P_{a}=\frac{1}{1+\exp(\frac{R_{b}-R_{a}+\alpha \left(I_{b}-I_{a}\right)+B\left(s_{b}-s_{a}\right)}{\sigma_{d}})} \end{array}$$

The values of *R*_*a*_ and *R*_*b*_ are specified as the observed means of the forced-choice outcomes of the respective options. *I*_*a*_ is specified such that if option *b* is more informative (i.e., when only one outcome has been observed for that option in unequal information games) then *I*_*b*_ − *I*_*a*_ = 1, whereas this value is made negative if option *a* is more informative. *I*_*b*_ − *I*_*a*_ = 0 within equal information games. The spatial variable (i.e., identifying the option being on the left vs. the right) is set to *s*_*b*_ − *s*_*a*_ = 1 when option *b* is on the right; this value is made negative when option *a* is on the right. Fitting this model to participants' behavior allows estimation of the values of the information bonus (α), decision noise (σ_*d*_), and spatial bias (*B*) that best account for patterns of decision-making at the individual level (here, this was accomplished using the gradient-based ‘fmincon’ function in MATLAB to find parameters that maximize the probability of participants' behavior under the model). These values can then be used as individual difference measures to assess how each may relate to other variables of interest. The information bonus is estimated separately for H1 and H6 conditions, and the difference in these two estimates (α_*H*6_ − α_*H*1_) is taken as the measure of directed exploration. The decision noise is estimated separately for H1 and H6 conditions as well as for equal and unequal information conditions. This allows calculation of two measures of random exploration – one for equal and one for unequal information conditions – by taking the difference between σ_*d*_ in H6 and H1 games for each condition. In this study, we did not perform further analyses on spatial bias estimates, which are included primarily to remove this as a confounding influence on estimating the parameters of interest.

#### General Reflectiveness Measures

To thoroughly characterize individual differences in general reflectiveness, we used three related measures of reflective cognition chosen to span different aspects of this construct and to evaluate convergent validity across different measurement approaches (i.e., self-reported attitudes vs. performance measures). These measures included the cognitive reflection test [CRT-7; (28)], the actively open-minded thinking scale [AOMTS; (30)], and the 2-subscale version of the comprehensive assessment of rational thinking [CART; (29)]. The CRT-7 asks seven short questions designed such that there is an immediately intuitive, but incorrect, answer, and a correct answer that, while not logically difficult, requires the individual to devote effortful cognitive resources instead of immediately choosing the intuitively appealing response. Example item:

“If it takes 5 machines 5 min to make 5 widgets, how long would it take 100 machines to make 100 widgets?” (intuitive answer: 100 min; correct answer: 5 min).

It tests the tendency to “stop and think” before immediately trusting one's intuition.

The AOMTS is a self-report scale which asks individuals to rate 30 statements, from 1 (strongly disagree) to 6 (strongly agree), which describe more or less reflective or “rational” attitudes. Example item:

“I like to gather many different types of evidence before I decide what to do.”

Higher scores indicate more open-minded, reflective attitudes.

The CART assesses vulnerability to various common reasoning biases that arise (in part) from insufficient engagement of reflective capacities. The 2-subscale version we used includes statistical and scientific reasoning problems. Example item:

“Dice game: Even numbers win and odd numbers lose on a die throw. The fair die has six sides, with three even and three odd numbers. Jan has thrown seven odd numbers in a row. What are her chances of throwing an even number on her next throw?” (correct answer: 3/6).

Higher scores indicate a greater tendency to engage effortful cognition and avoid common reasoning biases during problem-solving.

When assessing differences on these reflective cognition measures, it is important to first account for individual differences in general intelligence. To do so, we also asked participants to complete the 2-subscale Wechsler abbreviated scale of intelligence [WASI-II; (34)], a common measure of IQ.

### Analyses

To assess each of the hypothesized relations between exploration, cognitive reflection, and depression/anxiety symptoms, we ran JZS Bayes factor analyses with default prior scales in R [BayesFactor package (35, 36)] comparing null (intercept only) models to the space of models that included all combinations of main effects of age, sex, IQ, and the predictor of interest (and interactions between sex and the predictor of interest) on the relevant target variables. Interactions with sex were included because sex differences have been observed in both cognitive reflection measures (37–42) and symptoms of emotional disorders (43–47). Age was included due to previous work showing changes in exploratory behavior across childhood and adolescence (48, 49). Models included exploration measures as predictors of depression/anxiety symptoms, cognitive reflection measures as predictors of exploration measures, and cognitive reflection measures as predictors of depression/anxiety symptoms. To assess whether cognitive reflection measures and exploration measures accounted for shared vs. independent variance in symptoms, we ran further models including both types of measures as predictors of symptom measures in cases where both measures were predictors of symptoms in independent models.

When comparing models, a Bayes factor (BF) represents the ratio of the probability of the data under one model vs. another, indicting the relative evidence for different models. If *H*_0_ indicates the null hypothesis, *H*_1_ indicates the alternative hypothesis, and *d* indicates the data, then:


$$\begin{array}{l} BF=\;\frac{p\left(d|H_{1}\right)}{p\left(d|H_{0}\right)} \end{array}$$

A higher BF value indicates greater evidence for the alternative hypothesis; e.g., BF = 3 indicates three times as much evidence for the alternative hypothesis than for the null hypothesis. A BF < 1 instead indicates data are more likely under the null hypothesis; e.g., BF = 0.20 indicates that the data are five times more probable under a model that does not include an effect of interest. We adopt the guidelines described in Lee and Wagenmakers (50) for interpreting the strength of evidence provided by different BF values: BF = 1–3, poor/anecdotal evidence; 3–10, moderate evidence; 10–30, strong evidence, 30–100, very strong evidence, >100, extremely strong evidence. Empirical means and credible intervals for each variable in winning models were found by sampling from the posterior of the model, using Markov chain Monte-Carlo (MCMC) sampling for 10,000 iterations.

After identifying the winning models, we conducted *post-hoc* Pearson correlations to better interpret the strength of relationships between each predictor variable and each target variable. To better interpret findings regarding directed and random exploration, we also examined additional Horizon Task performance measures. This included the information bonus (α) and decision noise (σ_*d*_) parameters for H1 and H6 trials (see Figures 1D–F), total choice accuracy (i.e., number of overall free choices corresponding to the arm with the higher true mean value; see Figure 1G), and average reaction time for the first free choice on each trial. We also calculated the distance between information bonus values and their optimal values. This was motivated by inspection of scatterplots (shown in Figures 1H,I) relating exploration measures to choice accuracy, which suggested inverted-U effects for information bonus values for both H1 and H6 trials (IBH1 and IBH6). In each case, this was captured by the fact that a quadratic model fit the data better than a linear model (Akaike information criterion [AIC] = −606.78 vs. AIC = −507.69 for IBH1; AIC = −2209.45 vs. AIC = −2168.41 for IBH6). As expected theoretically, choice accuracy was highest for IBH1 values near 0. The empirically optimal value for IBH6 (based on the maximum of a quadratic model) was 6.78. Therefore, we computed absolute deviations from optimal values for both conditions (i.e., from 0 for IBH1 and 6.78 for IBH6) and used these values in some secondary analyses.

To provide an estimate of the power afforded by our sample size (*N* = 416) to detect relationships between variables of different effect sizes, we ran simulations sampling 416 datapoints from distributions with different true correlation values (100,000 simulations each) and calculated the probability with which those relationships were detected, based on a threshold of BF > 3. This revealed an approximately 80% probability of detecting a small effect size correlation of *r* = 0.165.

## Results

The descriptive statistics for all variables are shown in Tables 1, 2. These tables also include comparisons between males and females, motivated by the reliable sex differences found for these variables in previous work described above. As seen there, depression and anxiety were primarily within the subclinical range, but values indicative of moderate to severe symptoms (BDI > 20, STAI > 40) were also represented. STAI Trait scores were greater in females, while CRT-7, AOMTS, and CART scores were greater in males. Females showed faster first-choice RTs in the Horizon Task as well as moderate evidence for greater decision noise in H1 trials with unequal information. No sex differences were found in choice accuracy or measures of directed or random exploration. Two sample *t*-tests comparing students to non-students also did not reveal evidence for differences in any study measures (all BFs < 0.20, favoring the null hypothesis).

**Table 1:** Descriptive statistics and tests of sex differences for age, symptoms, and reflectiveness.

	**Total**	**Female**	**Male**	Usable Data (N)^*^	Effect of Sex^**^
Age	23.75 (5.61)	23.43 (5.49)	24.61 (5.84)	Female: 301 Male: 115 Total: 416	t(414) = −1.93 d = −0.19 BF = 0.71
BDI	7.31 (6.79)	7.65 (6.92)	6.4 (6.36)	Female: 301 Male: 115 Total: 416	t(414) = 1.69 d = 0.17 BF = 0.47
STAI State	30.12 (8.22)	30.21 (8.14)	29.87 (8.46)	Female: 300 Male: 115 Total: 415	t(413) = 0.38 d = 0.04 BF = 0.13
STAI Trait	36.55 (10.98)	37.51 (10.75)	34.06 (11.25)	Female: 300 Male: 115 Total: 415	t(413) = 2.89 d = 0.28 **BF** **=** **6.37**
WASI	108.37 (11.47)	107.92 (11.14)	109.55 (12.24)	Female: 300 Male: 115 Total: 415	t(413) = −1.3 d = −0.13 BF = 0.27
CART	10.52 (2.44)	10.1 (2.26)	11.62 (2.56)	Female: 299 Male: 114 Total: 413	t(411) = −5.91 d = −0.58 **BF > 100**
AOMTS	134.78 (13.44)	133.57 (12.98)	137.93 (14.16)	Female: 300 Male: 115 Total: 415	t(413) = −2.98 d = −0.29 **BF = 8.37**
CTR−7	2.65 (2.05)	2.35 (1.95)	3.44 (2.13)	Female: 291 Male: 109 Total: 400	t(398) = −4.84 d = −0.49 **BF > 100**

* *Not all data were available from all participants. We therefore report Ns for each measure*.

** *Results of Bayesian two-sample t-tests. Bayes factors (BFs) supporting a group difference with values greater than 3 are bolded. Effect sizes (Cohen's d) are also shown*.

**Table 2:** Descriptive statistics and tests of sex differences for Horizon Task variables.

	**Total**	**Female**	**Male**	Usable Data (N)^*^	Effect of Sex^**^
Dir. Exp.	6.26 (11.79)	5.79 (11.85)	7.48 (11.57)	Female: 301 Male: 115 Total: 416	t(414) = −1.3 d = −0.13 BF = 0.27
Info. Bonus H1	−0.73 (8.82)	−1.01 (9.16)	0 (7.86)	Female: 301 Male: 115 Total: 416	t(414) = −1.04 d = −0.1 BF = 0.2
Info. Bonus H6	5.53 (11.31)	4.78 (11.4)	7.47 (10.86)	Female: 301 Male: 115 Total: 416	t(414) = −2.18 d = −0.21 BF = 1.17
Rand. Exp. (Equal Info)	3.23 (6.37)	3.13 (6.4)	3.51 (6.32)	Female: 301 Male: 115 Total: 416	t(414) = −0.55 d = −0.05 BF = 0.14
Dec. Noise H1 (Equal Info)	4.4 (7.25)	4.69 (7.38)	3.64 (6.88)	Female: 301 Male: 115 Total: 416	t(414) = 1.33 d = 0.13 BF = 0.28
Dec. Noise H6 (Equal Info)	7.63 (7.78)	7.82 (7.65)	7.15 (8.12)	Female: 301 Male: 115 Total: 416	t(414) = 0.79 d = 0.08 BF = 0.16
Rand. Exp. (Unequal Info)	2.63 (6.51)	2.51 (6.76)	2.94 (5.82)	Female: 301 Male: 115 Total: 416	t(414) = −0.6 d = −0.06 BF = 0.14
Dec. Noise H1 (Unequal Info)	4.96 (6.74)	5.5 (7.17)	3.54 (5.2)	Female: 301 Male: 115 Total: 416	t(414) = 2.68 d = 0.26 **BF = 3.73**
Dec. Noise H6 (Unequal Info)	7.59 (7.12)	8.01 (7.38)	6.48 (6.28)	Female: 301 Male: 115 Total: 416	t(414) = 1.98 d = 0.19 BF = 0.78
Info. Bonus – Deviation from Optimal H1	5.71 (6.76)	6.21 (6.8)	4.4 (6.5)	Female: 301 Male: 115 Total: 416	t(414) = 2.46 d = 0.24 BF = 2.16
Info. Bonus – Deviation from Optimal H6	9.18 (6.7)	9.34 (6.82)	8.76 (6.39)	Female: 301 Male: 115 Total: 416	t(414) = 0.79 d = 0.08 BF = 0.16
First–Choice RT	1.46 (0.73)	1.38 (0.62)	1.67 (0.92)	Female: 301 Male: 115 Total: 416	t(414) = −3.69 d = −0.36 **BF = 76.3**
First–Choice RT H1	1.41 (0.78)	1.32 (0.67)	1.62 (0.97)	Female: 301 Male: 115 Total: 416	t(414) = −3.56 d = −0.35 **BF = 49.52**
First-Choice RT H6	1.51 (0.74)	1.44 (0.63)	1.72 (0.95)	Female: 301 Male: 115 Total: 416	t(414) = −3.51 d = −0.34 **BF = 41.73**
Choice Accuracy	0.23 (0.03)	0.22 (0.03)	0.23 (0.03)	Female: 301 Male: 115 Total: 416	t(414) = −2 d = −0.2 BF = 0.82
Choice Accuracy H1	0.8 (0.13)	0.79 (0.13)	0.82 (0.13)	Female: 301 Male: 115 Total: 416	t(414) = −1.9 d = −0.19 BF = 0.68
Choice Accuracy H6	0.13 (0.02)	0.13 (0.02)	0.13 (0.02)	Female: 301 Male: 115 Total: 416	t(414) = −1.83 d = −0.18 BF = 0.6

* *Not all data were available from all participants. We therefore report Ns for each measure*.

** *Results of Bayesian two-sample t-tests. Bayes factors (BFs) supporting a group difference with values greater than 3 are bolded. Effect sizes (Cohen's d) are also shown*.

### Initial Validity Analyses

Consistent with previous work, IBH6 values were greater than IBH1 values (*t*_(415)_ = 10.83, *d* = 0.53, BF > 100). Likewise, decision noise in H6 (DNH6) trials was also greater than in H1 (DNH1) trials in both equal (*t*_(415)_ = 10.34, *d* = 0.51, BF > 100) and unequal (*t*_(415)_ = 8.25, *d* = 0.40, BF > 100) information conditions (see Figures 1D,E). Plotting the proportion of optimal choices as a function of choice number showed the expected pattern in which (1) the lower mean option was chosen more often at the first free choice in H6 than H1 trials, and (2) performance improved with each subsequent choice in H6 trials (Figure 1G).

Intercorrelations between Horizon Task measures were consistent with expectations and supported the validity of model parameters. Decision noise estimates in all conditions showed negative correlations with choice accuracy (*r*s between −0.57 and −0.83, all BFs > 100) and first-choice RTs (*r*s between −0.15 and −0.30, BFs between 13.5 and > 100). First-choice RTs were positively correlated with choice accuracy (*r*s between 0.17 and 0.28, BFs between 57.5 and > 100). Random exploration scores for equal and unequal information conditions were both positively associated with directed exploration (*r*s = 0.175 and 0.183, BFs = 67.8 and > 100) and choice accuracy in H1 trials (*r*s = 0.21 and 0.18, BFs > 100). IBH1 values were negatively correlated with choice accuracy (*r*s between −0.16 and −0.25, BFs between 26.5 and > 100). Directed exploration was positively correlated with choice accuracy (*r*s between 0.22 and 0.24, all BFs > 100). Neither directed nor random exploration was associated with first-choice RTs (*r*s between−0.06 and 0.06, BFs between 0.12 and 0.26).

Cognitive reflectiveness measures were correlated in the expected directions: CART:CRT-7 (*r* = 0.66, BF > 100); CART:AOMTS (*r* = 0.37, BF > 100); CRT-7:AOMTS (*r* = 0.30, BF > 100). Each measure also showed the expected moderate correlation with WASI IQ scores (CART: *r* = 0.54, BF > 100; CRT-7: *r* = 0.54, BF > 100; AOMTS: *r* = 0.32, BF > 100).

Symptom measures were also correlated in the expected directions: STAI State:BDI (*r* = 0.60, BF > 100); STAI Trait:BDI (*r* = 0.80, BF > 100); STAI State:STAI Trait (*r* = 0.68, BF > 100).

### Exploration as a Predictor of Symptoms

**Figure 2:**
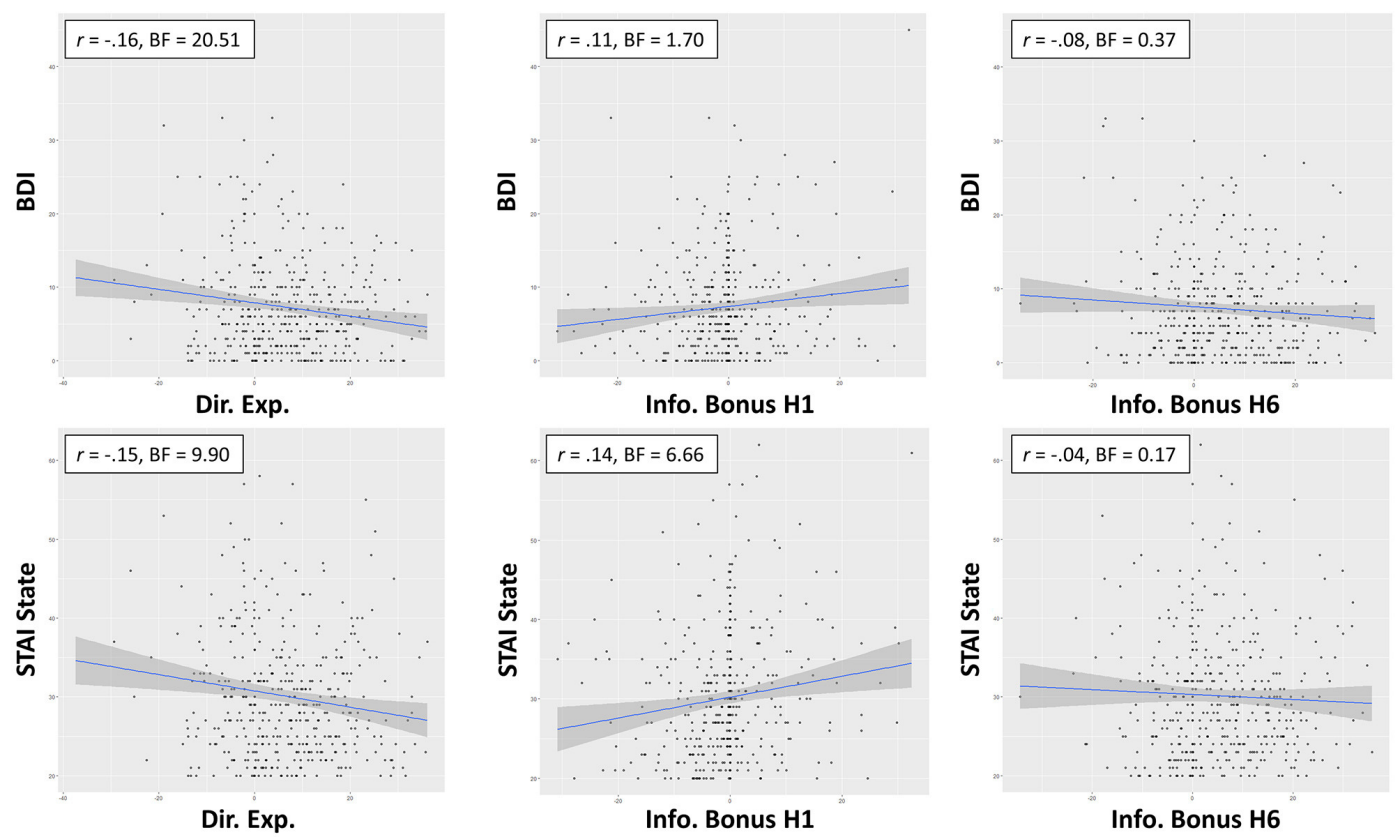
Zero-order relationships between directed exploration and depression (BDI) and state anxiety (STAI). Relationships between information bonus in H1 and H6 trials suggested this was explained in part by greater information seeking in H1 trials with more severe symptoms.

As shown in Table 3, the best models included directed exploration as a predictor of both STAI State scores (negative relationship; BF = 7.56 compared to an intercept-only model; moderate evidence) and BDI scores (negative relationship; BF = 16.62 compared to an intercept-only model; strong evidence; see Figure 2). STAI Trait scores were best explained by a model that only included sex.

**Table 3:** Exploration as a predictor of symptoms.

	**Dir. Exp**.	**Rand. Exp. (Equal Info)**	**Rand. Exp. (Unequal Info)**
Outcome Variable^*^			
STAI State	Best Model (M1): **Dir. Exp**. b = −0.1, CI = [−0.17, −0.03] M1/Intercept: **BF = 7.56** 2nd Best Model (M2): Dir. Exp. + Age M1/M2: BF = 2.84 N: - Female: 299 - Male: 115 - Total: 414	Best Model: Intercept only N: - Female: 299 - Male: 115 - Total: 414	Best Model: Intercept only N: - Female: 299 - Male: 115 - Total: 414
STAI Trait	Best Model (M1): Sex b = 1.67, CI = [0.53, 2.81] M1/Intercept: BF = 6.75 2nd Best Model (M2): Sex + Dir. Exp.^*^Sex M1/M2: BF = 1.28 N: - Female: 299 - Male: 115 - Total: 414	Best Model (M1): Sex b = 1.68, CI = [0.49, 2.86] M1/Intercept: BF = 6.75 2nd Best Model (M2): Sex + Rand. Exp. (Equal Info)^*^Sex M1/M2: BF = 6.42 N: - Female: 299 - Male: 115 - Total: 414	Best Model (M1): Sex b = 1.67, CI = [0.52, 2.84] M1/Intercept: BF = 6.75 2nd Best Model (M2): Sex + Rand. Exp. (Unequal Info) M1/M2: BF = 7.27 N: - Female: 299 - Male: 115 - Total: 414
BDI	Best Model (M1): **Dir. Exp**. b = −0.09, CI = [−0.14, −0.03] M1/Intercept: **BF = 16.62** 2nd Best Model (M2): Dir. Exp. + WASI M1/M2: BF = 1.67 N: - Female: 300 - Male: 115 - Total: 415	Best Model: Intercept only N: - Female: 300 - Male: 115 - Total: 415	Best Model: Intercept only N: - Female: 300 - Male: 115 - Total: 415

*For ease of inspection, the model predictors and BFs consistent with a priori hypotheses have been bolded*.

* *Not all data were available from all participants. We therefore report Ns for each measure*.

To interpret these results, we ran several *post-hoc* analyses. We first ran correlations between BDI/STAI State scores and IBH1 and IBH6 values separately. Correlations were weakly positive with IBH1 (STAI State: *r* = 0.14, BF = 6.66; BDI: *r* = 0.11, BF = 1.7) but absent in IBH6 (STAI State: *r* = −0.04, BF = 0.17; BDI: *r* = −0.08, BF = 0.37), suggesting that the negative relationship between directed exploration and BDI/STAI State scores was primarily accounted for by greater information-seeking in H1 trials, as opposed to lower information-seeking in H6 trials. As the relationship between IBH1 and choice accuracy reported above was negative, this suggested that stronger symptoms were associated with a suboptimal strategy. However, choice accuracy did not appear directly related to these symptoms in IBH6 (STAI State: *r* = −0.08, BF = 0.39; BDI: *r* = −0.11, BF = 1.29). Symptoms were also not associated with deviation from optimal IBH1 or IBH6 values (*r*s between 0.02 and 0.08, BFs between 0.12 and 0.46) or with first-choice RTs (*r*s between −0.03 and −0.10, BFs between 0.14 and 0.98).

Although the zero-order correlations between BDI/STAI were only significant for information bonus at H1, it was also possible that IBH6 values could predict differences in depression/anxiety after accounting for differences in IBH1 (and/or that these variables interacted). To test this, we compared BFs for models including IBH1, IBH6, and/or their interaction as predictors of BDI/STAI. The winning model for predicting BDI (BF = 4.77 relative to an intercept only model) included both a positive relationship with IBH1 (b = 0.12, CI = [0.04 0.19]) and a negative relationship with IBH6 (b = −0.07, CI = [−0.13 −0.2]), but no interaction. This model had a BF = 3.11 relative to a model removing the effect of IBH6. The winning model for predicting STAI State (BF = 6.26 relative to an intercept only model) similarly included both a positive relationship with IBH1 (b = 0.16, CI = [0.07 0.25]) and a negative relationship with IBH6 (b = −0.07, CI = [−0.14 0.0]), but no interaction. This model had a BF = 1.06 relative to a model removing the effect of IBH6. Thus, depression and anxiety levels were both also associated with reduced IBH6 after accounting for individual differences in baseline (Horizon 1) information bonus values, although the explanatory value of IBH6 was poor in the case of anxiety.

Random exploration values were not included in the winning models predicting any symptom measure (see Table 3). There was also no relationship between symptoms and decision noise in H1 or H6 trials (*r*s between −0.02 and 0.07, BFs between 0.11 and 0.29), except for possible anecdotal evidence of a positive relationship between BDI and decision noise in H6 trials with unequal information (*r* = 0.12, BF =2.56).

### Cognitive Reflection as a Predictor of Symptoms

**Figure 3:**
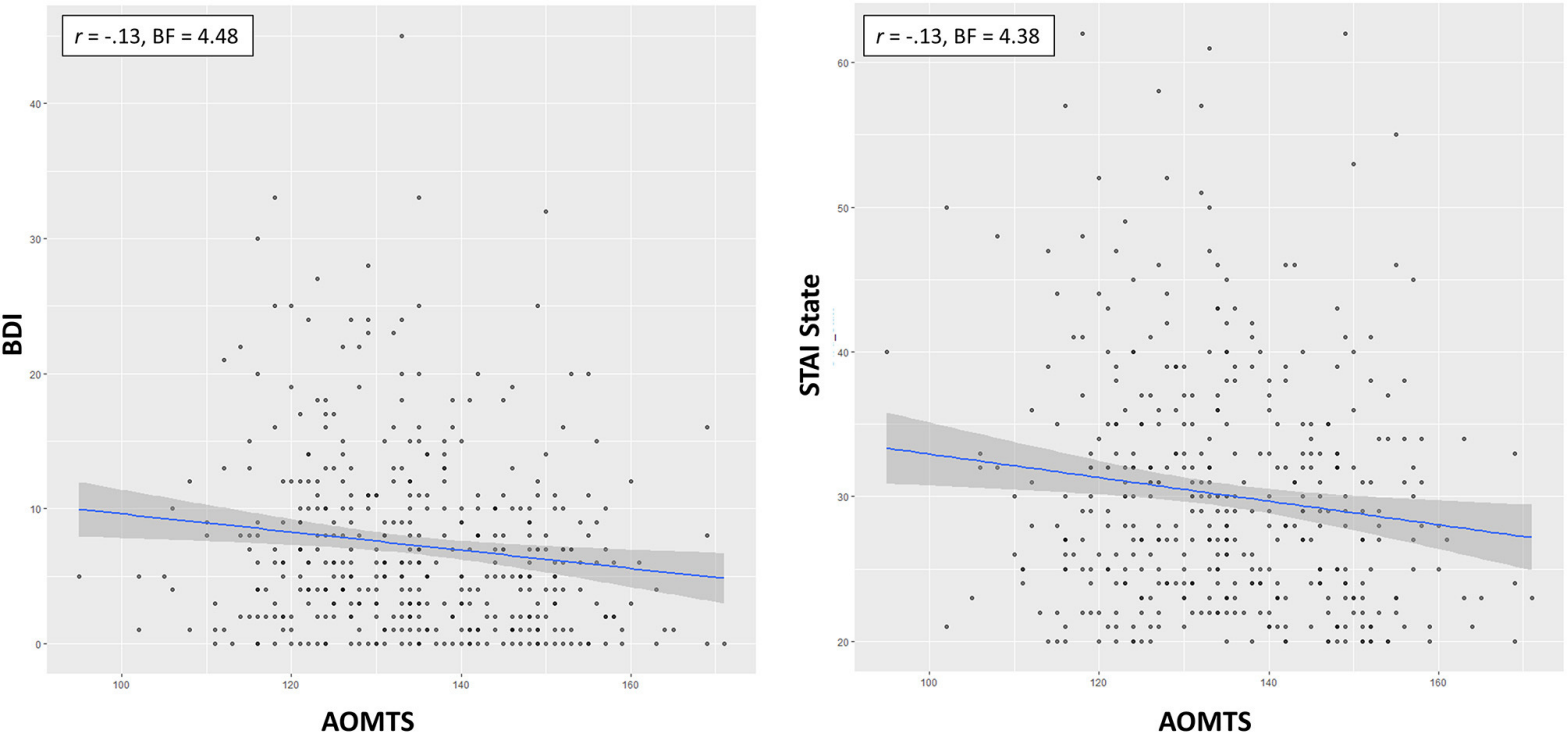
Relationships between self-reported reflectiveness (AOMTS) and depression (BDI) and state anxiety (STAI). Greater reflectiveness was associated with lower symptom levels.

As shown in Table 4, the best models included AOMTS as a predictor of STAI State scores and BDI scores (negative relationship in both cases; BFs = 3.3 and 3.7, respectively; see Figure 3). STAI Trait scores remained best accounted for by a model including only sex as a predictor.

**Table 4:** Cognitive reflection as a predictor of symptoms.

	**CART**	**CRT**	**AOMTS**
Outcome variable^*^			
STAI State	Best Model: Intercept only N: - Female: 298 - Male: 114 - Total: 412	Best Model: Intercept only N: - Female: 290 - Male: 109 - Total: 399	Best Model (M1): **AOMTS** b = −0.08, CI = [−0.13, −0.02], M1/Intercept: **BF = 3.30** 2nd Best Model (M2): AOMTS + AOMTS^*^Sex M1/M2: BF = 2.70 N: - Female: 299 - Male: 115 - Total: 414
STAI Trait	Best Model (M1): Sex b = 1.66, CI = [0.5, 2.81] M1/Intercept: BF = 6.05 2nd Best Model (M2): Sex + CART M1/M2: BF = 6.81 N: - Female: 298 - Male: 114 - Total: 412	Best Model (M1): Sex b = 1.64, CI = [0.43, 2.82] M1/Intercept: BF = 4.78 2nd Best Model (M2): Sex + CRT M1/M2: BF = 3.25 N: - Female: 290 - Male: 109 - Total: 399	Best Model (M1): Sex b = 1.68, CI = [0.51, 2.82] M1/Intercept: BF = 6.75 2nd Best Model (M2): Sex + AOMTS M1/M2: BF = 2.20 N: - Female: 299 - Male: 115 - Total: 414
BDI	Best Model (M1): WASI b = −0.06, CI = [−0.12, −0.01] M1/Intercept: BF = 1.02 2nd Best Model (M2): CART M1/M2: BF = 1.20 N: - Female: 298 - Male: 114 - Total: 412	Best Model (M1): WASI b = −0.07, CI = [−0.13, −0.01] M1/Intercept: BF = 1.71 2nd Best Model (M2): WASI + Sex M1/M2: BF = 2.98 N: - Female: 290 - Male: 109 - Total: 399	Best Model (M1): **AOMTS** b = −0.06, CI = [−0.11, −0.02] M1/Intercept: **BF = 3.71** 2nd Best Model (M2): AOMTS + WASI M1/M2: BF = 2.62 N: - Female: 299 - Male: 115 - Total: 414

*For ease of inspection, the model predictors and BFs consistent with a priori hypotheses have been bolded*.

* *Not all data were available from all participants. We therefore report Ns for each measure*.

### Cognitive Reflection as a Predictor of Exploration

**Figure 4:**
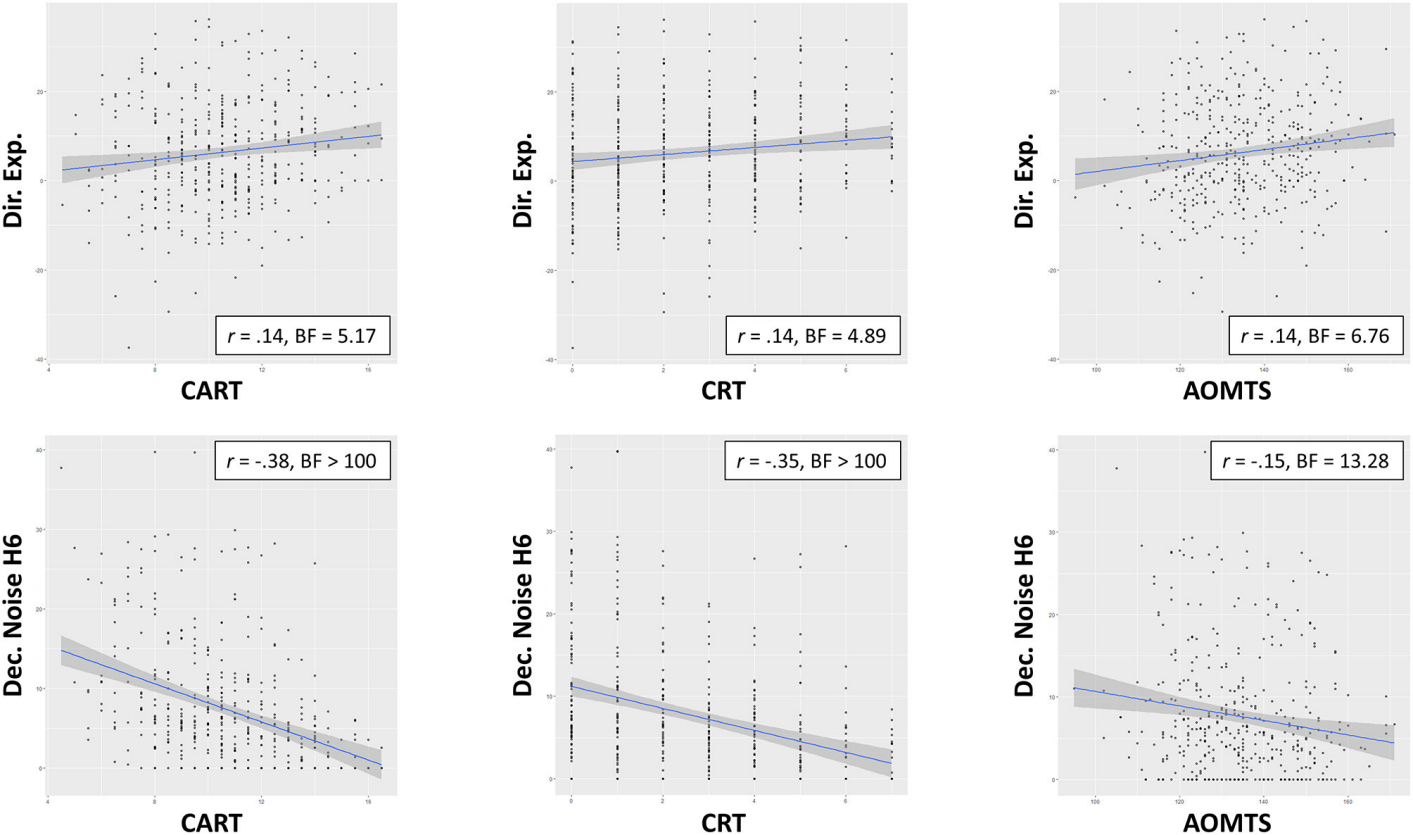
Relationships between self-reported reflectiveness (CART. CRT-7, AOMTS), directed exploration, and decision noise. Greater reflectiveness was associated greater exploration and reduced decision noise.

As shown in Table 5, when CART was included as the predictor of interest for directed exploration, the winning model included effects of CART (positive relationship) and an interaction between CART and sex (stronger relationship between directed exploration and CART in males; BF = 5.94; moderate evidence; see Figure 4). The main effect of the CART was most important, as the data became 26.22 times less probable when it was removed, but only 1.43 times less probable when the interaction was removed. When assessing CRT-7 as the predictor of interest for directed exploration, the winning model only included CRT-7 (positive relationship; BF = 3.79; moderate evidence). Similarly, when assessing AOMTS as the predictor of interest for directed exploration, the winning model only included AOMTS (positive relationship; BF = 5.12; moderate evidence).

**Table 5:** Cognitive reflection as a predictor of exploration.

	**CART**	**CRT**	**AOMTS**
Outcome Variable^*^			
Dir. Exp.	Best Model (M1): CART + CART^*^Sex - CART: b = 0.77, CI = [0.29, 1.25]. **VI: BF = 26.22** - CART^*^Sex: b = −0.52, CI = [-1,−0.03]. VI: BF = 1.43 M1/Intercept: **BF = 5.94** 2nd Best Model (M2): CART M1/M2: BF = 1.43 N: - Female: 298 - Male: 114 - Total: 412	Best Model (M1): **CRT** b = 0.75, CI = [0.2, 1.31] M1/Intercept: **BF = 3.79** 2nd Best Model (M2): WASI M1/M2: BF = 1.98 N: - Female: 290 - Male: 109 - Total: 399	Best Model (M1): **AOMTS** b = 0.12, CI = [0.03, 0.2] M1/Intercept**: BF = 5.12** 2nd Best Model (M2): AOMTS + WASI M1/M2: BF = 1.2 N: - Female: 299 - Male: 115 - Total: 414
Rand. Exp. (Equal Info)	Best Model: Intercept only N: - Female: 298 - Male: 114 - Total: 412	Best Model: Intercept only N: - Female: 290 - Male: 109 - Total: 399	Best Model: Intercept only N: - Female: 299 - Male: 115 - Total: 414
Rand. Exp. (Unequal Info)	Best Model: Intercept only N: - Female: 298 - Male: 114 - Total: 412	Best Model: Intercept only N: - Female: 290 - Male: 109 - Total: 399	Best Model: Intercept only N: - Female: 299 - Male: 115 - Total: 414

*VI, Variable importance. This is the BF of the best model compared to a model in which the relevant predictor is removed. For ease of inspection, the model predictors and BFs consistent with a priori hypotheses have been bolded*.

* *Not all data were available from all participants. We therefore report Ns for each measure*.

To interpret these results, we did several *post-hoc* analyses. We first ran correlations between CART/CRT-7/AOMTS and IBH1 and IBH6 values separately. For CART and CRT-7, positive correlations were present with IBH6 (CART: *r* = 0.16, BF = 20.18; CRT-7: *r* = 0.17, BF = 49.82). AOMTS was not correlated with IBH6, and no reflectiveness measures were correlated with IBH1 (*r*s between −0.11 and 0.06, BFs between 0.13 and 1.23). This suggested that greater reflectiveness led to greater increases in exploration on H6 trials. We next examined whether reflectiveness measures predicted deviations from optimal IBH1 and IBH6 values – finding that higher CART and CRT-7 scores predicted less deviation from optimal IBH1 values (CART: *r* = −0.25, BF > 100; CRT-7: *r* = −0.27, BF > 100) and that higher CRT-7 scores also predicted less deviation from optimal IBH6 values (*r* = −0.17, BF = 29.58). We also assessed how cognitive reflectiveness values related to choice accuracy and first-choice RTs. Choice accuracy was higher with greater reflectiveness scores on all measures (CART: *r* = 0.44, BF > 100; CRT-7: *r* = 0.40, BF > 100; AOMTS: *r* = 0.20, BF > 100), and those with higher reflectiveness scores also showed slower RTs (CART: *r* = 0.24, BF > 100; CRT-7: *r* = 0.23, BF > 100; AOMTS: *r* = 0.27, BF > 100).

The effect of CART/CRT-7 on IBH6 was not accounted for by differences in IQ when models were compared that also included age, sex, or WASI scores (winning models only included CART or CRT-7, with BFs of 16.18 and 38.06 relative to an intercept-only model, respectively). The same was true of relationships between choice accuracy and all three measures of reflectiveness, where winning models included both reflectiveness measures and WASI as an additional predictor (BFs > 100 in all cases). CART and CRT-7 were more important, with data becoming > 100 times less probable when each was removed, while removing the WASI only made the data 2.18 and 12.52 times less probable, respectively. However, WASI was more important than AOMTS, with data becoming > 100 times less probable when WASI was removed, but only 1.14 times less probable when AOMTS was removed.

As shown in Table 5, random exploration was not predicted by CART, CRT-7, or AOMTS in the best models. However, there was very strong evidence for negative relationships between CART/CRT-7 and decision noise in both H1 (DNH1) and H6 (DNH6) trials (*r*s between −0.25 and −0.39, all BFs > 100; see Figure 4). There was also evidence for relationships between decision noise and AOMTS (*r*s between −0.15 and −0.21, BFs between 13.28 and > 100), with the exception of DNH6 on trials with unequal information.

As shown in Table 6, the relationships between decision noise and CART/CRT-7 were not accounted for by differences in IQ. Specifically, these measures were not removed as predictors of DNH1 or DNH6 values when WASI, age, or sex were considered as additional predictors, and BFs strongly favored models with these reflectiveness measures included compared to when they were removed. AOMTS was retained as a predictor in the winning model for DNH1 in equal information trials, but all other DN values were better explained by WASI scores.

**Table 6:** Cognitive reflection as a predictor of decision noise.

	**CART**	**CRT**	**AOMTS**
**Outcome variable**			
Dec. Noise H1 (Unequal Info)	Best Model (M1): **CART + WASI** - CART: b = −0.64, CI = [−0.93, −0.35], **VI: BF > 100** - WASI: b = −0.08, CI = [−0.14, −0.01], VI: BF = 2.57 M1/Intercept: **BF > 100** 2nd Best Model (M2): CART M1/M2: BF = 2.57 N: - Female: 298 - Male: 114 - Total: 412	Best Model (M1**): CRT + WASI** - CRT: b = −0.71, CI = [−1.05, −0.35], **VI: BF > 100** - WASI: b = −0.08, CI = [−0.15, −0.02], VI: BF = 3.41 M1/Intercept: **BF > 100** 2nd Best Model (M2): CRT M1/M2: BF = 3.41 N: - Female: 290 - Male: 109 - Total: 399	Best Model (M1): Sex + WASI - Sex: b = 0.81, CI = [0.13, 1.49], VI: BF = 1.97 - WASI: b = −0.15, CI = [−0.2, −0.09], VI: BF > 100 M1/Intercept: BF > 100 2nd Best Model (M2): WASI M1/M2: BF = 1.85 N: - Female: 299 - Male: 115 - Total: 414
Dec. Noise H6 (Unequal Info)	Best Model (M1): **CART** b = −0.7, CI = [−0.98, −0.43] M1/Intercept: **BF > 100** 2nd Best Model (M2): CART + WASI M1/M2: BF = 2.81 N: - Female: 298 - Male: 114 - Total: 412	Best Model (M1): **CRT** b = −0.83, CI = [−1.16, −0.5] M1/Intercept: **BF > 100** 2nd Best Model (M2): CRT + WASI M1/M2: BF = 3 N: - Female: 290 - Male: 109 - Total: 399	Best Model (M1): WASI b = −0.12, CI = [−0.17, −0.06] M1/Intercept: BF > 100 2nd Best Model (M2): Sex + WASI M1/M2: BF = 2.04 N: - Female: 299 - Male: 115 - Total: 414
Dec. Noise H1 (Equal Info)	Best Model (M1): **CART + WASI** - CART: b = −0.95, CI = [−1.25, −0.64], **VI: BF > 100** - WASI: b = −0.07, CI = [−0.14, −0.01], VI: BF = 1.18 M1/Intercept: **BF > 100** 2nd Best Model (M2): CART M1/M2: BF = 1.18 N: - Female: 298 - Male: 114 - Total: 412	Best Model (M1): **CRT + WASI** - CRT: b = −1, CI = [−1.38, −0.64], VI: **BF > 100** - WASI: b = −0.09, CI = [−0.16, −0.03], VI: BF = 4.05 M1/Intercept: **BF > 100** 2nd Best Model (M2): CRT + Age + WASI M1/M2: BF = 3.2 N: - Female: 290 - Male: 109 - Total: 399	Best Model (M1): **AOMTS + WASI** - AOMTS: b = −0.06, CI = [−0.12, −0.01], **VI: BF = 2.49** - WASI: b = −0.16, CI = [−0.22, −0.1], VI: BF > 100 M1/Intercept: **BF > 100** 2nd Best Model (M2): AOMTS + WASI + AOMTS^*^Sex M1/M2: BF = 1.14 N: - Female: 299 - Male: 115 - Total: 414
Dec. Noise H6 (Equal Info)	Best Model (M1): **CART + WASI** - CART: b = −0.92, CI = [−1.25, −0.59], VI: **BF > 100** - WASI: b = −0.1, CI = [−0.17, −0.02], VI: BF = 4.02 M1/Intercept: **BF > 100** 2nd Best Model (M2): CART M1/M2: BF = 4.02 N: - Female: 298 - Male: 114 - Total: 412	Best Model (M1): **CRT + WASI** - CRT: b = −0.93, CI = [−1.35, −0.52], VI: **BF > 100** - WASI: b = −0.13, CI = [−0.2, −0.05], VI: BF = 25.07 M1/Intercept: **BF > 100** 2nd Best Model (M2): CRT + Sex + WASI M1/M2: BF = 5.31 N: - Female: 290 - Male: 109 - Total: 399	Best Model (M1): WASI b = −0.2, CI = [−0.26, −0.14] M1/Intercept: BF > 100 2nd Best Model (M2): AOMTS + WASI M1/M2: BF = 4.07 N: - Female: 299 - Male: 115 - Total: 414

*VI, Variable importance. This is the BF of the best model compared to a model in which the relevant predictor is removed. For ease of inspection, the model predictors and BFs consistent with a priori hypotheses have been bolded*.

* *Not all data were available from all participants. We therefore report Ns for each measure*.

### Cognitive Reflection and Exploration as Independent Predictors of Symptoms

The only cases in which both cognitive reflection measures and exploration measures predicted symptom measures in the winning models were with AOMTS and directed exploration as predictors of STAI State and BDI scores. When including models with both as possible predictors of STAI State, each was retained in the winning model (BF = 14.58 relative to an intercept-only model), suggesting they account for unique variance in anxiety symptoms. Directed exploration was a more important predictor, as the full model had a BF of 4.42 compared to a model removing this variable, while it only had a BF of 1.93 compared to a model removing AOMTS. When including models with both as possible predictors of BDI, each was retained in the winning model (BF = 20.24 relative to an intercept-only model), suggesting they also account for unique variance in depressive symptoms. Directed exploration was a more important predictor, as the full model had a BF of 5.46 compared to a model removing this variable, while it only had a BF of 2.08 compared to a model removing AOMTS.

### *Post-hoc* Correlations and Sex Differences

**Figure 5:**
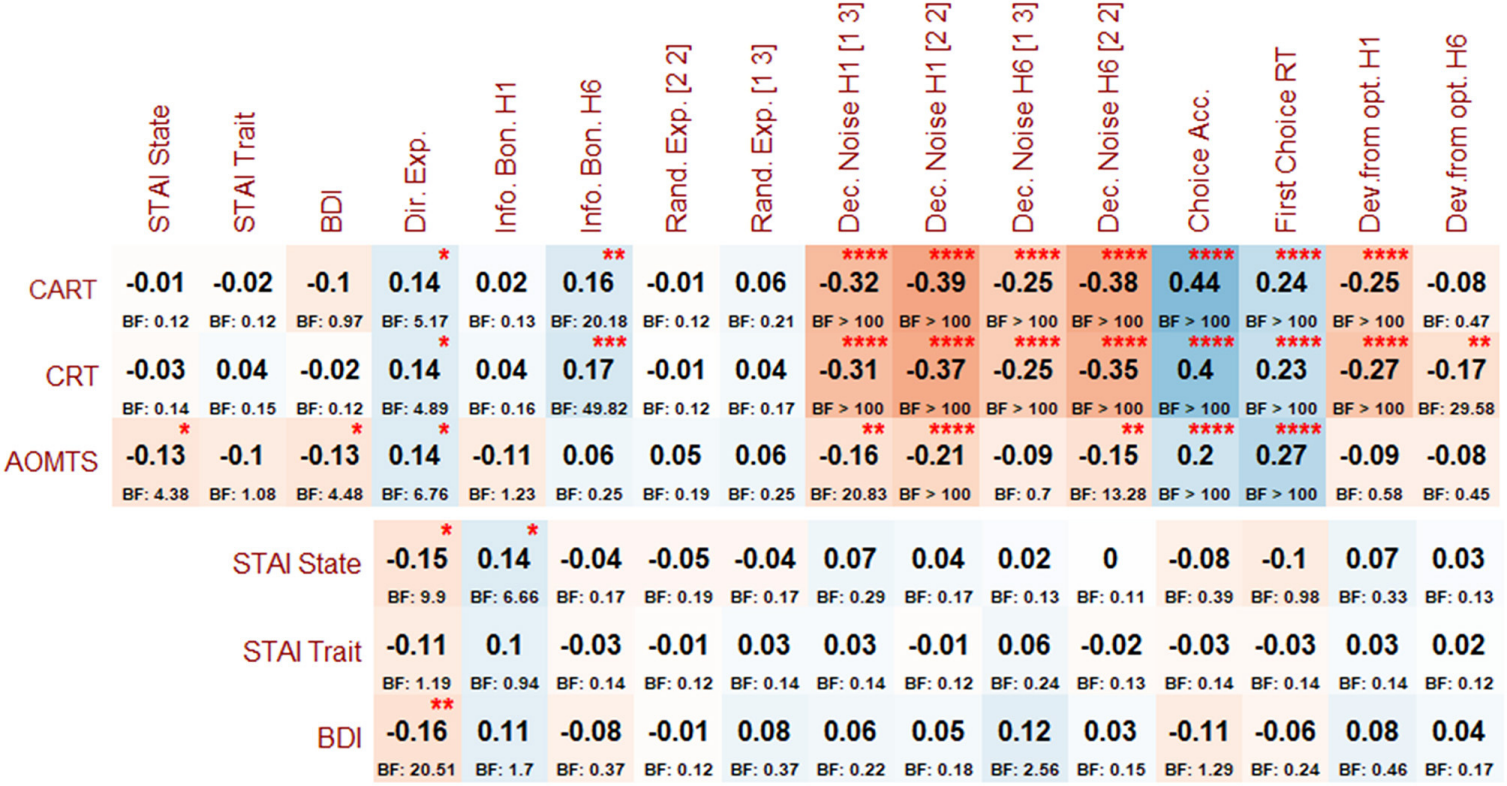
*Post-hoc* correlations (and associated BFs) between reflectiveness measures, symptoms, and Horizon Task measures. Asterisks indicate BFs greater than 3, 10, 30, and 100, per conventional cutoffs for levels of evidence from moderate to extremely strong.

For the interested reader, *post-hoc* correlations between all variables are provided in Figure 5. These correlations recapitulated each of the primary results presented above.

Given the sex differences observed across several variables, we also ran exploratory correlations separately by sex to see if this might offer additional insights. These supplementary analyses suggested that observed relationships with symptoms were driven more strongly by females and that relationships between reflectiveness and exploration were more strongly driven by males (see Supplementary Figure 1). However, the difference in sample sizes between males and females limits the interpretability of these results, and they were not hypothesized. We mention them here simply as preliminary findings that could support generation of future hypotheses.

## Discussion

In this study, we tested several predictions about the relationship between cognitive reflectiveness, exploratory behavior, and symptoms of depression/anxiety. We first tested the prediction that differences in depression and anxiety would be predicted by differences in exploratory behavior. This was motivated by two competing hypotheses: (1) a negative relationship would be observed between exploration and symptoms, consistent with maladaptive avoidance behavior, and (2) a positive relationship would instead be observed, consistent with sustained uncertainty about optimal choice and resulting in reduced reward-seeking. We also tested the subsequent prediction that differences in exploratory behavior and symptoms could be accounted for by differences in general reflectiveness.

In support of the first hypothesis, and in line with prior work linking anhedonia to reduced exploration (26), we found that directed exploration was lower in those with more severe depression and state anxiety symptoms. This is consistent with the possibility that symptoms of depression and anxiety are maintained by maladaptive exploratory drives.

However, further analyses indicated that these results were explained in large part by greater baseline exploration in H1 games (i.e., when exploration is not beneficial) as opposed to only being due to lower exploration in H6 games. Specifically, a lower information bonus in H6 only accounted for differences in depression and anxiety after controlling for differences in H1, and this additional explanatory power was moderate for depression but poor for anxiety. This suggests that greater symptoms were also associated with an increased drive to reduce ambiguity – that is, to reduce uncertainty at the potential cost of reduced task performance. This is consistent with previous work suggesting that depression and anxiety are associated with intolerance of uncertainty [e.g., see (51)] – but is less consistent with the hypothesis that reduced information-seeking prevents adaptive learning and maintains symptoms. However, it is important to consider that the current version of the Horizon Task is framed with respect to wins (i.e., reward maximization). Avoidance behavior might be more specifically associated with reduced exploration in the context of avoiding negative outcomes. As such, it remains an open question whether these same results would be found if this task were instead framed in terms of losses [i.e., where participants begin with a certain amount of money or points and must choose the option that will lead to smaller losses; e.g., see (18, 52)].

Although we find a negative relationship between symptoms and directed exploration, our findings linking elevated symptoms to greater ambiguity aversion share some similarity to the greater exploration in depression (25) and trait anxiety (24) observed in previous work. However, there are important differences worth considering. First, these studies have used different tasks that do not distinguish directed and random exploration in the way we have done here. Second, the above-mentioned study on depression focused on comparing groups with low vs. high symptom severity using a different measure of depression. Third, our findings are with respect to state anxiety, while those found previously pertain to trait anxiety (for which we did not find significant results). Future studies will therefore be necessary to assess the consistency between these different findings.

With respect to our subsequent hypothesis about reflectiveness, we observed the predicted relationship in which directed exploration was positively associated with all three cognitive reflection measures. One measure of reflectiveness (AOMTS) was also negatively associated with depression and state anxiety. Notably, all results remained present when considering IQ as another explanatory variable in the space of possible models, suggesting that differences in cognitive ability did not account for the relationships between reflectiveness measures and either symptoms or exploration. In further model comparison including both AOMTS and directed exploration as symptom predictors, each predictor was retained in the best model. Omission of directed exploration from the winning model also led to greater reductions in model evidence than omission of AOMTS, suggesting exploration had higher importance as an explanatory variable. This pattern of results therefore does not support a mediation-like relationship (i.e., reflectiveness and exploration each accounted for unique variance in symptoms). Indeed, while the relationship between directed exploration and symptoms was explained in part by greater exploration in H1 trials (as mentioned above), the relationship with two of the reflectiveness measures (CART and CRT-7) was primarily explained by greater exploration in H6 trials, and the third (AOMTS) was explained by lower exploration in H1 trials (i.e., each of which would be beneficial to maximizing long-run reward). Both CART and CRT-7 also specifically predicted lower deviations from optimal levels of exploration.

These results are consistent with the idea that greater trait reflectiveness promotes greater (and more adaptive) levels of information-seeking – offering one potential explanation for why some individuals engage in directed exploration more than others. Interestingly, one recent study using transcranial magnetic stimulation (TMS) found evidence for a causal role of right frontopolar cortex in controlling directed (but not random) exploration (especially in the long horizon condition) (53), and this brain region has also been linked to other reflective processes such as prospection and farsighted planning (54), as well as the management of competing goals (55). This could therefore suggest that directed exploration emerges (in part) as a result of a broader set of reflective processes focused on prospective decision-making in consideration of proximal vs. distal objectives.

In contrast, we found evidence against relationships between random exploration and both reflectiveness and symptoms. However, we observed that higher IQ and higher reflectiveness were associated with less decision noise in both H1 and H6 games within the Horizon Task. Model comparison also suggested that reflectiveness accounted for a greater (and unique) proportion of the variance in decision noise relative to IQ. This was part of a broader pattern in which reflectiveness measures were also associated with slower RTs and higher choice accuracy, and decision noise was associated with faster RTs and lower choice accuracy. Together, this suggests that less reflection led to faster choices that were more random (across all task conditions) and therefore led to reduced performance. Measures of reflectiveness are therefore not sensitive to changes in randomness between task conditions (i.e., random exploration), but they offer an explanation for the degree to which individuals think through their choices before responding.

This study has a number of strengths and limitations. One strength is the large sample size, which afforded sufficient power to detect small effect sizes. Another was the use of Bayesian analytic approaches that allowed for model comparison and assessment of evidence both for and against the null hypothesis. For example, in addition to supporting some of our primary hypotheses, we also find moderate evidence against relationships between trait anxiety and most other study variables, as well as evidence against relationships between random exploration and either reflectiveness or depression/anxiety symptoms. One limitation is that the sample had a greater number of females than males, which limited interpretability of potential effects of sex. For example, exploratory analyses suggested that some relationships we observed may differ in males and females. However, interactions with sex were most often not included in the winning models predicting symptoms or exploratory behavior. A larger number of males could have provided more evidence for such interactions or, alternatively, could have led such suggestive patterns to disappear. Thus, future work could follow up on this to examine this possibility further. Another limitation is that we used a shorter version of the Horizon Task (80 games), while previous studies have used longer versions (up to 320 games). This smaller number of games could have made our parameter estimates less reliable, and therefore added noise to the data. This raises the possibility that stronger relationships may have been found if parameters were fit to larger amounts of choice data. Finally, our study focused on young adults and 68.5% were college students. This could be important as explore-exploit behavior is known to change with age in childhood (49) and adolescence (48), which are key periods for the development of mental health. Students could also differ from non-students in the same age range (although we did not find evidence for differences between students and non-students on the measures included here).

To conclude, in this study we found that less directed exploration was associated with greater levels of anxiety and depression, and that this was explained in part by a type of ambiguity aversion in which information-seeking was elevated in contexts where it would not benefit subsequent choices – and may relate to broader patterns of intolerance of uncertainty. We also found that differences in directed exploration were accounted for by trait differences in cognitive reflectiveness, and that reflectiveness was also lower in those with greater depression/anxiety symptoms; yet, reflectiveness and exploration accounted for unique variance in symptoms. Reflectiveness levels further accounted for decision noise, reaction times, and choice accuracy. Together, these results shed light on the mechanisms underlying information-seeking behavior and how they may contribute to symptoms of emotional disorders. Although many effect sizes were small, they also suggest the possibility that reflectiveness and exploration could represent clinically relevant mechanisms in the subset of individuals who show low levels of these tendencies – consistent with the need to develop individualized precision medicine approaches within computational psychiatry. Future research should replicate this work and could extend it by examining the role of reflectiveness in other psychiatric contexts as well as how exploration is affected in situations that require avoidance of negative outcomes.

## Data Availability Statement

The raw data supporting the conclusions of this article will be made available by the authors upon request, without undue reservation.

## Ethics Statement

All study procedures involving human participants were reviewed and approved by the University of Arizona Institutional Review Board (Protocol #1607696724). All participants provided their written informed consent to participate in this study.

## Author Contributions

RS helped with study design and data collection, performed the primary analyses, created figures, and wrote the initial draft of the manuscript. ST helped with literature review, data analysis, and figure creation and also edited the manuscript. AC helped with literature review and figure creation and also edited the manuscript. SW and RW each helped with task design, initial data processing, and manuscript editing. MP helped with data collection, data organization, and manuscript editing. WK acquired funding for the study, helped design the study, and edited the manuscript. All authors contributed to the article and approved the submitted version.

## Funding

The study was supported by funding from the U.S. Army Medical Research and Development Command (W81XWH-16-1-0062) to WK. RS was supported by the William K. Warren Foundation and the National Institute of General Medical Sciences (P20GM121312). RW was supported by the National Institute on Aging (R01AG061888).

## Conflict of Interest

The authors declare that the research was conducted in the absence of any commercial or financial relationships that could be construed as a potential conflict of interest.

## Publisher's Note

All claims expressed in this article are solely those of the authors and do not necessarily represent those of their affiliated organizations, or those of the publisher, the editors and the reviewers. Any product that may be evaluated in this article, or claim that may be made by its manufacturer, is not guaranteed or endorsed by the publisher.

## Supplementary Material

Supplementary Figure 1 *Post-hoc* correlations (and associated BFs) between reflectiveness measures, symptoms, and Horizon Task measures. This is analogous to Figure 5 in the main text, but examines males and females separately – and suggests that observed relationships between directed exploration and symptoms were driven by females while relationships between directed exploration and reflectiveness measures were driven by males. Asterisks indicate BFs greater than 3, 10, 30, and 100, per conventional cutoffs for levels of evidence from moderate to extremely strong.
