# Supplementary figures and images for: Lower Levels of Directed Exploration and Reflective Thinking Are Associated With Greater Anxiety and Depression
Source: Front Psychiatry. 2022 Jan 7;12:782136. doi: 10.3389/fpsyt.2021.782136 (PMC8808291; doi:10.3389/fpsyt.2021.782136)

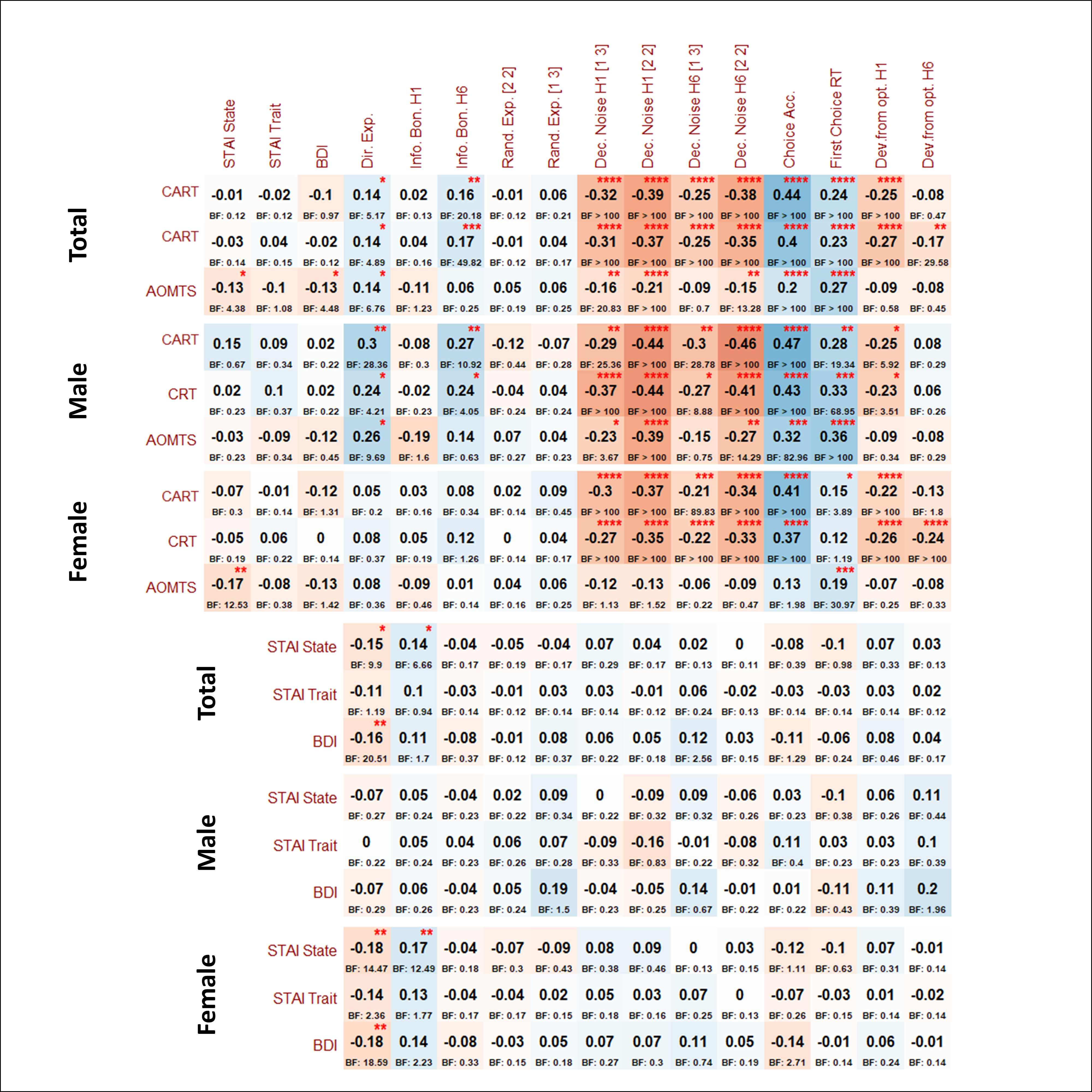

Supplement: Supplementary Figure 1 — Post-hoc correlations (and associated BFs) between reflectiveness measures, symptoms, and Horizon Task measures. This is analogous to Figure 5 in the main text, but examines males and females separately – and suggests that observed relationships between directed exploration and symptoms were driven by females while relationships between directed exploration and reflectiveness measures were driven by males. Asterisks indicate BFs greater than 3, 10, 30, and 100, per conventional cutoffs for levels of evidence from moderate to extremely strong. [file Image_1.TIF]
